# Aromatic Perspectives: An In-Depth review on Extracting, influencing Factors, and the origins of raisin aromas

**DOI:** 10.1016/j.fochx.2024.101285

**Published:** 2024-03-15

**Authors:** Hafiz Umer Javed, Yuan-sen Liu, Jun-guang Hao, Faisal Hayat, Murtaza Hasan

**Affiliations:** a College of Food Engineering, Beibu Gulf University 535011, Qinzhou, Guangxi, China; b Guangxi College and University Key Laboratory of High-Value Utilization of Seafood and Prepared Food in Beibu Gulf, College of Food Engineering, Beibu Gulf University 535011, Qinzhou, China; c College of Horticulture, Zhongkai University of Agriculture and Engineering, Guangzhou 510225, China; d College of Chemistry and Chemical Engineering, Zhongkai University of Agriculture and Engineering, Guangzhou 510225, China

**Keywords:** Raisin, Drying, Volatile organic compounds, Aroma, SPME

## Abstract

Raisins, derived from dried grapes, represent a valuable commodity rich in secondary metabolites, particularly volatile organic compounds (VOCs). The primary objective of this review is to identify the VOCs that are influencing the aromatic profile of raisins to improve consumer preferences. However, extensive research has been done to optimize grape drying methods for different raisin attributes. In the context of this review, an in-depth investigation of published literature revealed the extraction of over 120 VOCs from raisins using SPME. Furthermore, we explored factors shaping raisin aroma and the sources of VOC generation. This review aims to pinpoint research gaps and provide an opportunity for future developments in studying raisins' aroma. This involves integrating advanced analytical techniques, examining processing method impacts, and considering consumer perception to enhance the overall understanding of raisin aromas. The outcomes are anticipated to provide valuable insights for the industry and the scientific community.

## Highlights

• 120 VOCs are listed as a raisin volatile.
• SPME is the most sophisticated technique for extracting VOCs in raisins.
• MR, UFAO and terpenoids are the primary factors influencing the raisin aromas.
• Raisin aromas are mainly influenced by drying processing and pre-treatments.

## Graphical abstract

**Figure:**
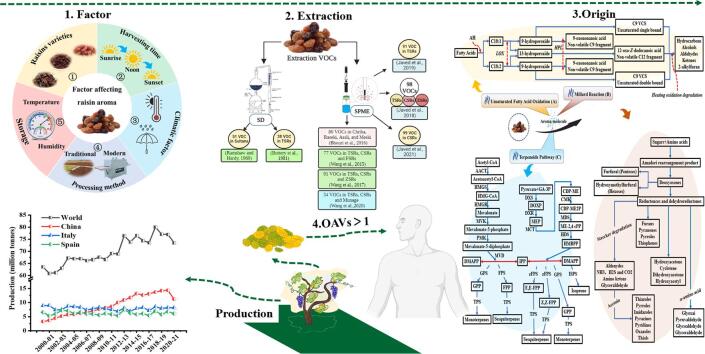
Factors affecting, extraction method and origin of raisin volatile organic compounds.

## 1 Introduction

Grapes are a non-climacteric berry fruit, generally growing in a cluster on deciduous woody vines. Grapes belong to the 'Vitaceae' family and are produced worldwide. The worldwide production of grapes increased per year by a million tons (MT), as shown in Supplementary Fig. 1. In the year 2021, China topped the list for grape production with 11.2 million metric tons, while Italy (8.15 MT) and Spain (6.07 MT) secured the 2nd and 3rd positions, respectively, according to the Food and Agriculture Organization of the United Nations (2023). Grapes can be consumed as fresh fruit or used in various products such as juice, jelly, vinegar, seed oil, wine, and raisins (Venkitasamy et al., 2019).

Raisins are dehydrated grapes produced in grape-growing regions and consumed throughout the world as a snack, as well as used in baking, brewing, and cooking. In terms of dried fruits production, raisins, and currants were in the top position with the production of 1,262 thousand metric tons (TMT) following dates (836 TMT), prunes (253 TMT), apricots (160 TMT), and figs (121 TMT) were reported by the global statistical organization (INC, 2016). According to Statista report, China is the second biggest raisin-producing (2.20 TMT) country after Turkey (Shahbandeh, 2022). The major production area for raisin is Turpan, Xinjiang, which accounts for nearly 75 % of the total production of China (Khiari et al., 2018). More than 20 different kinds of varieties were planted for making raisins. Among these, 'Thompson Seedless (TS) is a prominent variety because it is more suitable for processing and cultivated up to 85–90 % of the total grape area in the Turpan state of China (Inouye et al., 2016). A number of raisin varieties were produced based on consumer preferences, including small, medium, and large, as well as seeded and seedless grapes in green, black, brown, blue, purple, and yellow color.

Grapes, being seasonal fruit, are processed into raisins because they are not always available in the market. There are two primary classifications of raisin drying technologies: traditional and modern approaches. Traditionally, raisins are produced under the sun or in the air (Wang et al., 2015). In order to increase the rate of dehydration and ensure a higher quality of the raisins, various modern drying techniques have been used, including microwave, vacuum pulsed, oven, and infrared drying (Wang et al., 2016). The first raisins were thought to have been produced in the Near East by simply digging the grapes in the sand. Drying grapes significantly increased their shelf life and produced a high sugar content, making them ideal energy sources for laborers (Carughi et al., 2008). Furthermore, it inhibits the growth of mold, yeast, and bacteria, slows down enzymatic degradation, and inactivates basic physical and biochemical activities by eliminating the water until the appropriate level of moisture is obtained. (Khiari et al., 2018). Modern and traditional drying techniques significantly affect the changes in VOCs that contribute to raisin aroma (Torres et al., 2015, Wang et al., 2015, Wang et al., 2017, Wang et al., 2017).

Raisins are comprised of sugar about 72 % by weight (Winkler et al., 1974), which mainly consist of fructose and glucose. They also contain higher contents of antioxidants, such as in apricots and prunes, but the concentration of vitamin C is lower than in fresh grapes. Raisins are found to be cholesterol-free dry fruit. The National Nutrient Database from the United States Department of Agriculture (USDA, 2018) reported the nutritional values of the raisin seedless (*Vitis vinifera*) per 100 g are mentioned in Table 1.

**Table 1:** Nutritionals values of *Vitis vinifera* per 100 g.

Principle	Nutrient Value		Minerals	Nutrient Value
Energy	299 Kcal		Selenium	0.6 µg
Carbohydrates	79.18 g		Zinc	0.22 mg
Protein	3.07 g		**Vitamins**	**Nutrient Value**
Total Fat	0.46 g		Pyridoxine	0.0174 mg
Cholesterol	0 mg		Riboflavin	0.125 mg
Dietary Fiber	3.7 g		Thiamin	0.106 mg
**Minerals**	**Nutrient Value**		Vitamin A	0 IU
Calcium	50 mg		Vitamin C	2.3 mg
Copper	0.318 mg		Vitamin E	0.12 mg
Iron	1.88 mg		Vitamin K	3.5 µg
Magnesium	7 mg		**Electrolytes**	**Nutrient Value**
Manganese	0.299 mg		Sodium	1 mg
Phosphorus	101 mg		Potassium	749 mg

According to the National Nutrient Database from the United States Department of Agriculture (USDA, 2018).

The aroma of raisins is an integral sensory quality that affects customer acceptance. It is the result of the accumulation of several VOCs, and the composition varies according to the variety and species (Schwab et al., 2008), geographical regions (Feng et al., 2022), vintage (Yuan & Qian, 2015), drying methods (Torres et al., 2015, Wang et al., 2015, Wang et al., 2017, Wang et al., 2017), storage conditions and packaging materials (Kaack & Christensen, 2010). Approximately 120 VOCs are thought to make up raisins volatile profile, including free-form and glycosidically bound compounds. These VOCs belong to various chemical classes (aldehydes, esters, acids, alcohols, ketones, terpenes, and furans) and have a variety of flavor characteristics (fruity, green, floral, roasted, fatty, and chemical). The major sources of raisin VOCs include fresh grapes, Maillard reaction (MR), oxidation of unsaturated fatty acid, carotenoids, and lipids (Javed et al., 2018, Javed et al., 2019, Yuan and Qian, 2015).

Numerous reviews have been published on topics such as raisin drying methods, phytochemical attributes, and the associated health benefits (Khiari et al., 2018; Williamson and Carughi, 2010, Wong et al., 2013). Grapes drying has been extensively studied and there are many fascinating instances in the literature, we discovered that there is not a comprehensive review that addresses raisin aromas and the sources of VOCs such as UFAO, MR, terpenoid, and carotenoids pathways. In this review, we have explored factors that affect the raisin aromas including grape varieties, harvesting time, drying methods, climatic factors, processing methods (traditional and modern), and storage. Aroma is a key trait that has gained more attention these days. Aroma research has markedly advanced in several domains due to the rapid advancement of science and technology, particularly using Gas chromatography-Mass spectrometry (GC–MS) and other analytical tools. In this overview, the components of raisin aroma and the process by which distinct aroma compounds are produced were briefly examined, followed by a discussion of the variables influencing the volatile nature of aroma compounds and their biosynthetic pathways.

## 2 Extraction of raisin volatiles

**Fig. 1:**
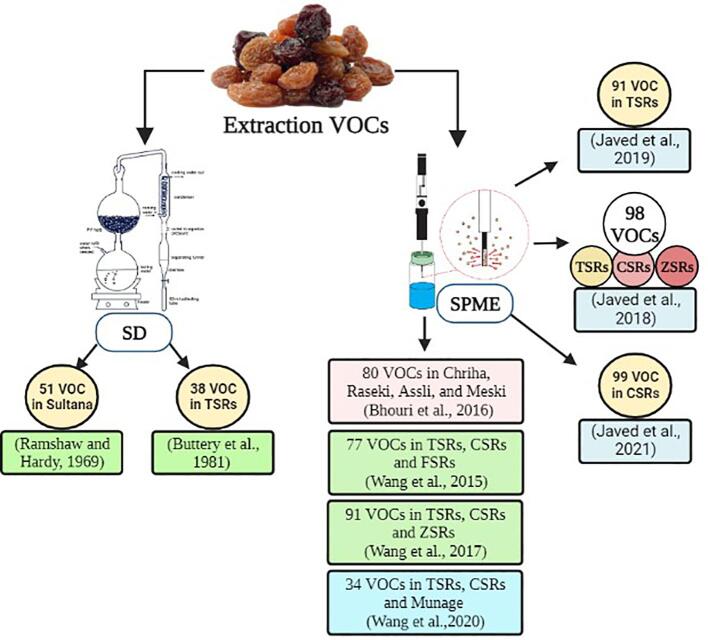
Identified raisin volatile compounds from different researchers (Bhouri et. al., 2016; Buttery et al., 1981, Javed et al., 2018, Javed et al., 2019, Javed et al., 2021, Ramshaw and Hardy, 1969, Wang et al., 2015, Wang et al., 2017, Wang et al., 2020, Wang et al., 2017, Wang et al., 2020) through distillation and SPME extraction method.

The identified raisin VOCs were extracted via the distillation process and the solid phase micro-extraction (SPME) technique is depicted in Fig. 1. In earlier research, the volatile components of raisins were isolated and extracted using simultaneous distillation extraction and steam distillation (Buttery et al., 1981, Joulain and Fourniol, 1990, Ramshaw and Hardy, 1969). Ramshaw & Hardy (1969), utilized steam distillation (a technique that uses heat to vaporize the VOCs, which are then condensed and collected) to characterize 51 VOCs in sultana raisins, whereas only 38 VOCs through simultaneous distillation extraction (in this technique, volatile organic chemicals were separated from complex liquid matrices using a combination of solvent extraction and steam distillation) was discovered in Thompson seedless raisins (Buttery et al., 1981). Palma & Taylor (2001) used carbon dioxide, at high pressure and high temperature to extract 5-hydroxymethyl-2-furaldehyde from raisins and found a quantity of 0.128 mg/g. Overall 67 VOCs have been found in previous research, and numerous significant volatile components are still missing due to the limitations of analysis techniques (Guiné et al., 2010). Afterward, SPME (a technique that involves collecting the VOCs present in the headspace above a sample of raisins, and trapping them on a solid-phase microextraction fiber) was used for the first time and 77 VOCs were extracted from three raisin cultivars (Wang et al., 2015). SPME is an inexpensive, easy-to-operate, relatively solvent-free, and sensitive technique as compared to others (Abdulra’uf & Tan, 2015; Silva et al., 2015). Furthermore, a number of studies were carried out, and the SPME technique was used to identify more than 100 raisin volatiles in different varieties during drying and storage (Javed et al., 2018, Javed et al., 2019, Javed et al., 2021; Tesi & Lenzi, 1998; Thakur et al., 1996, Wang et al., 2017, Wang et al., 2020, Wang et al., 2017, Wang et al., 2020, 2021). The SPME was found to be the most effective extraction method that was significant in the isolation of VOCs from raisins. Further work needs to be explored on a sophisticated technique that contributed a major role in extraction.

## 3 Factor affecting raisin aroma

**Fig. 2:**
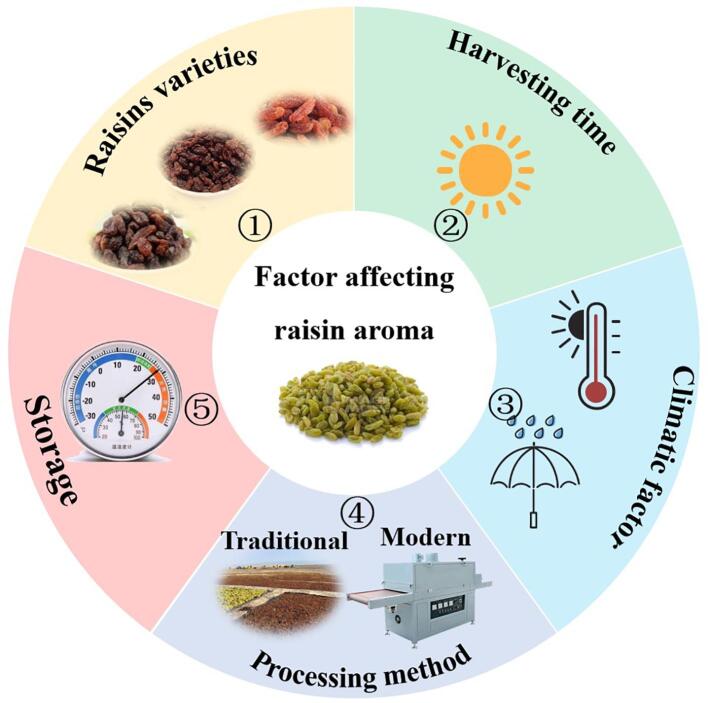
Raisin varieties, harvesting time, climatic factors, processing method, and storage affected raisin aroma.

The following factors such as grape variety, harvesting time, climatic, vintage, pre-treatment, processing method, and post-harvest handling and storage conditions (Fig. 2) have been identified to impact the quality and aroma profile of raisins, as detailed in Table 2 (Buttery, 2010, Wang et al., 2017, Wang et al., 2017). Raisins can be classified into different categories based on several factors, including grape cultivar attributes (such as size, color, and shape), the method of drying (such as green, golden bleached, sulfur bleached, and lexia), place of origin (like Patras, Malaga, Vostizza, Pyrgos, Symrne, and Valencia), size grading (including grade A, B, C, and substandard), as well as consumer preferences (like seeded, loose, and layer variations) (USDA, 2023).

**Table 2:** Factors affecting the quality and aroma of raisins.

	Product	Varieties	Results	Reference
***Raisin Varieties***				
Thompson Seedless (TS) Flame Seedless (FS) Crimson Seedless (CS)	Raisin		A total of 75, 72, and 74 VOCs were found in TS, FS, and CS raisins, respectively Floral and fruit aromas in CSRs > FSRs > TSRs Roasted aromas FSRs > CSRs > TSRs	Wang et. al., 2015
Thompson Seedless (TS) Centennial seedless (CS) Zixiang seedless (ZS)	Raisin		A total of 77, 80, and 90 VOCs were found in TS, CS, and ZS raisins, respectively Floral and fruit aromas in ZSRs > CSRs > TSRs Roasted aromas CSRs > ZSRs > TSRs Green aromas CSRs > ZSRs > TSRs	Wang et. al., 2017
Thompson Seedless (TS) Centennial seedless (CS) Zixiang seedless (ZS)	Raisin		A total of 81, 94, and 98 VOCs were found in TS, FS, and CS raisins, respectively Floral aromas in ZSRs > CSRs > TSRs Fruit aromas in ZSRs ≥ CSRs > TSRs Roasted aromas CSRs > ZSRs > TSRs Green aromas CSRs > ZSRs > TSRs	Javed et. al., 2018

***Harvesting Time***				
Local harvest schedule (LHS) 10 days after LHS (10-LHS) 20 days after LHS (20-LHS) 30 days after LHS (30-LHS)	Raisin	Paycamy	30-LHS increased 30 % the raisin yields as compared to LHS. 30-LHS has greater TSS (62.8 °Brix) and yield (2.14 kg) than the other harvests. Waste decay was also higher in 30 days-LHS	Arzani et al., 2009
20 days before LHS (20-BLHS) 10 days before LHS (10-BLHS) Local harvest schedule (LHS) 10 days after LHS (10-ALHS) 20 days after LHS (20-ALHS)	Raisin	Thompson seedless Manik Chaman 2A Clone Sonaka, Merbein Seedless	Maximum drying rate and weight average were recorded in 20-ALHS. Sensory attributes like color, texture, flavor, and overall acceptability scored higher in 20-ALHS, with values of 3.97, 3.89, 3.87, 4.09, and 3.96, respectively. Quality parameters such as TSS/TA, ascorbic acid, and sugar content increased in all raisin varieties with each harvest, peaking in 20-ALHS.	Venkatram et al., 2015
3 September 2020 (D0) 10 September 2020 (D7) 17 September 2020 (D14) 24 September 2020 (D21) 1 October 2020 (D28) 8 October 2020 (D35) 15 October 2020 (D42)	Grapes	Cabernet Sauvignon Cabernet Gernischet Cabernet Franc Merlot	The highest sugars/acids ratio was reached on D21 for Merlot and D28 for the other three varieties. Carbonyl compounds decreased consistently over the harvesting period. Free-form alcohols, esters, and terpene concentrations increased during ripening and decreased in overripe stages.	Gu et al., 2022

***Climatic Factor***				
45–50; airflow rates of 750 fpm	Raisin	Thompson Seedless Flame Seedless	Produced better color and sweet taste as compared to sun-dried raisins	Gee, 1980
40 °C + lyophilization 70, 65, and 60 °C + lyophilization, 70 °C + lyophilization	Raisin	Bezsemenné Perlette Vrboska Jakubské, Beauty seedless	40 °C + lyophilized had the best sensory quality in terms of smoothness, gloss, color, odor, chewability, juiciness, flavor, and overall impression as compared to the higher temperature	Langová et al., 2020
30 °C 40 °C 50 °C	Raisin	Zicui	Raisins dried at 50 °C had the highest levels of glucose, fructose, malic acid, shikimic acid, and succinic acid. Drying at 40 °C preferred the accumulation of various phenols while at 30 °C favored the accumulation of anthocyanins, flavonoids, and flavanols. Raisins produced at 30 °C had higher levels of volatile ketones and acids, with 2,6-dimethyl-4-heptanone content reaching 70.34 μg/L, significantly higher than in other raisins.	Chen et al., 2022
20 °C 30 °C Relative humidity 70 % or 90 %	Nut	Torreya grandis	A total of 56 VOCs were produced Alcohol and aldehyde levels varied significantly with temperature treatments, while terpene and alkane content were greatly affected by humidity treatments. *d*-limonene, the main terpene (63.0–90.8 %), was notably increased by high humidity.	Hu et. al., 2022

***Processing Method***				
Dipped raisins dried in racks Undipped raisins dried under the Sun	Raisin	Sultana	Dipped grapes dried in 2 weeks, while undipped grapes took 5 weeks to dry. Grapes dried under racks turned golden brown, while those dried under the sun turned dark brown. A total of 51 VOCs were identified, with 11 being unknown. Undipped sultanas contained higher levels of furfural and related compounds compared to dipped sultanas.	Ramshaw & Hardy, 1969
Sun-dried	Raisin	Thompson Seedless Centennial seedless Zixiang seedless	Identified 91 free and bound form VOCs ZSRs excelled with strong floral and fruity notes from decanal, rose oxide, linalool, geraniol, and *β*-damascenone.	Wang et. al., 2017
Dipping-Sun-dried (PSD) Dipping-Air-dried (PAD)	Raisin	Thompson Seedless Centennial seedless Zixiang seedless	A total of 98 VOCs were identified. The PAD drying method enhanced fruity and floral fragrances, while PSD treatment intensified fatty and roasted aromas.	Javed et. al., 2018
Sun-dried Air-dried	Raisin	Thompson Seedless	A total of 89 and 88 free-form compounds were identified in air-dried and sin-dried raisins, respectively. Air-dried raisins had floral and fruity aromas, whereas sun-dried raisins featured fatty, roasted, and chemical aromas.	Javed et. al., 2019
Microwave dried Freeze-dried Oven-dried 45 °C Oven-dried 60 °C Shade dried	Dried Strawberry	Strawberry	Shade air-dried, frozen, freeze-dried, and oven-dried 45 ◦C samples retained more of the fruity and sweet aromas of strawberries, representing more than 68 % of the total aroma intensity. In contrast, microwave-drying resulted in a significant loss of fruity esters, while oven-drying at 60 °C showed a moderate loss.	Abouelenein et al., 2021
Integrated freeze drying (IFD) Conventional freeze drying (CFD) Hot-air drying (AD)	Lemon	Lemon	22 VOCs were detected during the drying processes. After IFD, CFS, and AD, dried samples lost 7, 7, and 6 compounds, respectively, compared to fresh samples. The total loss rates of volatile compounds in the dried samples were 82.73 % in CFD, over 71.22 % in IFD, and over 28.78 % in AD.	Hu et al., 2023
***Storage***				
0, 3, 6, 9, 12, 15, 18 and 21 months	Tea	Sampo Sambu Samyl	Storage time impacted VOC content, with 15 VOCs disappearing partially or completely during processing or storage. Tea from Sampo and Sambu cultivars stored in plastic and aluminum bags maintained satisfactory flavor and VOC content for at least 3 months post-processing. In contrast, tea from Samyl had an unpleasant grassy off-flavor in all samples.	Kaack and Christensen, 2010
0, 3, 6 and 9 months	Raisin	Thompson Seedless Centennial seedless Zixiang seedless	UFAO and grape-derived compounds primarily added fruity, floral, and green aromas, which decreased in volatility over time. The levels of MR, CR, and EMP volatiles were significantly higher in the third month of storage.	Javed et. al., 2018
0, 3, 6, 9, and 12 months	Raisin	Thompson Seedless	Except for the chemical aroma, the fruity, floral, herbaceous, and roasted aromatic series intensified over the storage period, becoming more pronounced at 12 months.	Javed et. al., 2019

### 3.1 Raisins varieties

Different grape varieties contain unique VOCs that contribute to their specific aromas. Raisins are generally produced from four grape varieties (TS, Black Corinth, Muscat, and Sultana). Overall, 90 % of Thompson Seedless Raisins (TSRs) are produced in the United States (Bhat et al., 2006). For the production of raisins, more than 20 different grape cultivars are grown in China, including Beauty Seedless, Centennial Seedless (CS), Crimson Seedless, Flame Seedless (FS), Horse Milk, Hamburg, Muscat, Thomson Seedless, Ruby Seedless, etc. Among these, the Flame Seedless raisin (red variety), TS (green variety), and Crimson Seedless raisin (red variety) are the major varieties for raisin production (Wang et al., 2015).

Different raisin varieties produced different kinds of aromas and were characterized into different classes like green, fruity, floral, fatty, and roasted. Wang et al., 2017, Wang et al., 2017 experimented on three different types of seedless raisins: TS, CS, and Zixiang seedless (ZS). They found that Centennial seedless raisins (CSRs) had higher levels of floral, fruity, green, and roasted aromas than TSRs because of the presence of 2-pentylfuran, benzeneacetaldehyde, (*E*)-2-nonenal and 3-ethyl-2,5-dimethyl pyrazine. The Zixiang seedless raisins (ZSRs) exhibited more pronounced floral and fruity aromas compared to TSRs and CSRs while displaying reduced intensity of green and roasted aromas. Furthermore, a comprehensive study was performed during the storage of raisins for 0, 3, 6, and 9 months. A total of 81, 94, and 98 VOCs were quantified in TSRs, CSRs, and ZSRs, respectively. The aroma profile revealed that floral notes were the dominant characteristic in ZSRs, while CSRs exhibited noticeable roasted and herbaceous flavors (Javed et al., 2018). However, different varieties can be distinguished by their distinctive fragrances due to the specific generation or intensity of volatiles.

### 3.2 Harvesting time

Harvesting of fresh grapes is the initial step for the production of raisins. The best harvesting time is on a warm and sunny day when they are high in sugar content and have no surface moisture. Subsequently, ripening is an important factor for harvesting, described as the development of a waxy layer on grape skin, softening, turnover of color (according to variety), and changing the taste from sour to sweet (Jin et al., 2017). The harvesting time has a significant impact on the quality of raisins, particularly due to variations in the levels of soluble solids. Harvesting time and total soluble solids (TSS) vary according to the raisin varieties and environmental conditions. The three cultivars most commonly used to make raisins such as TS, FS, and Crimson ceedless, were harvested with 20–22 °Brix of TSS (Wang et al., 2015). The grapes that were harvested before ripeness had a high level of acidity, which ultimately led to the undesired raisin product. The yields and quality of raisins significantly improved when grapes were harvested at their optimal ripeness (Sharma et al., 2013). Accordingly, harvesting the grapes at the appropriate physiological maturity improves the plumpness, juiciness, and appealing wrinkled texture of raisins.

Arzani et al. (2009) conducted a study on four different harvesting times; in the first harvest the grapes were under the local harvest schedule (LHS), and then three further harvests were carried out with a ten-day gap. The results showed that the fourth harvest (30 days after LHS) had greater TSS (62.8 °Brix) and yield (2.14 kg) than the other harvests. Additionally, it takes less drying time to prepare raisins. In another study, the grape cultivars including TS, Manik Chaman, 2A Clone, Sonaka, and Merbein Seedless were harvested at five different intervals: 20 days before LHS, 10 days before LHS, LHS, 10 days after LHS, and 20 days after LHS. The findings demonstrated that the maximum rate of drying was achieved by raisins made from late-ripened grapes (20 days after LH) and a significant raisin recovery rate (26.20 %) was recorded in late-harvested TS grapes. Further, the sensory characteristics such as color, texture, flavor, and general acceptability with scores of 3.97, 3.89, 3.87, 4.09, and 3.96, respectively, as well as quality indicators like TSS/TA, ascorbic acid, and sugar content were increased in all raisin cultivars with each subsequent harvest date, reaching their greatest levels in the late harvest (Venkatram et al., 2015). Grapes that are harvested at different stages of ripeness will have different levels and types of volatile components. Therefore, to identify the optimal harvesting time, Gu et al. (2022) conducted a study on four grape varieties (Cabernet Sauvignon, Cabernet Gernischet, Cabernet Franc, and Merlot) harvested at seven different times from 3 September to 15 October, with 7-day intervals. They concluded that the peak sugars/acids ratio was achieved on day 21 for Merlot and on day 28 for the other three varieties. As ripening progresses, carbonyl compounds decrease while free-form alcohols, esters, and terpenes increase until the overripe stage. Additionally, bound-form aromas reach their peak on October 8th. This study suggests that optimal aroma maturity in tested grape varieties occurs later than the sugars/acids ratio in Ningxia, emphasizing the importance of aroma in grape ripening. Late harvesting enhances raisin quality and sensory attributes. Future research should explore the impact of harvesting time on raisin VOCs and aroma profiles.

### 3.3 Climatic factor

Grapevines are grown all over the world in a variety of climatic regions that offer a suitable environment for growing superior grapes and their products (raisins and wines). Climatic factors including temperature, humidity, light, and wind speed have a significant impact on the quality of raisins during the drying of grapes. The ideal temperature for drying grapes is between 40 and 45 °C, while low and high temperatures have an impact on raisins by delaying the drying process and changing their color, respectively (Sharma & Somkuwar, 2014). Higher humidity leads raisins to darken in color, and when these conditions persist for a while, raisins start to rot (Wang et al., 2016). In order to improve the color of the raisins, it is best to choose locations where the wind blows swiftly and with appropriate sunlight.

In earlier studies, two raisin varieties such as TS and FS were dried at 45–50 °C with airflow rates of 750 fpm responsible for better color and sweet taste as compared to sun-dried raisins (Gee, 1980). Langová and their Co. used five grape varieties including Bezsemenné, Perlette, Vrboska, Beauty seedless, and Jakubské that were dried under four different methods at various temperatures: drying at 40 °C, combination drying (70, 65, and 60 °C), drying at 70 °C, and lyophilizations. They found that those grapes dried at low temperatures (40 °C and lyophilized) had the best sensory quality in terms of smoothness, gloss, color, odor, chewability, juiciness, flavor, and overall impression as compared to the higher temperature (Langová et al., 2020). Chen and their colleagues have found 44 free-form VOCs in *Zicui* raisins that were dried at temperatures from 30 °C to 50 °C. The study revealed that raisins dried at 30 °C exhibited higher accumulations of acids and ketones. Specifically, the concentration of 2,6-dimethyl-4-heptanone, a compound known for its sweet odor, was significantly higher in raisins dried at 30 °C (measuring 70.34 μg/L) compared to raisins dried at 40 °C and 50 °C (Chen et al., 2022). Additionally, the interaction impact of temperature (20 °C and 30 °C) and relative humidity (70 % and 90 % RH) on the aroma profile of Torreya grandis nuts during drying was assessed, and both temperature and humidity were found to be effective. Particularly, terpenes were identified as the primary aromatic compounds, exhibiting a notable increase under conditions of elevated humidity. This augmentation can be attributed to the facilitation of *d*-limonene synthesis, a major constituent of terpene compounds, comprising a substantial proportion ranging from 63 % to 90.8 % (Hu et. al., 2022). The quality of the aroma is significantly influenced by temperature and humidity, which are important climatic factors. The majority of the research on raisins has focused on their quality parameter and sensory attributes. Further study is needed to identify the VOCs that are produced at various temperatures and to optimize the humidity for a richer aroma.

### 3.4 Processing method

The process of drying grapes is one of the most essential tasks for extending shelf life and minimizing financial losses. Furthermore, it improves the drying rate, as well as produces good quality products with distinctive aromas (Javed et al., 2018). The various methods that are being used to make raisins, such as traditional, modern, and chemical treatment, can also impact the VOCs present in the final product.

#### 3.4.1 Traditional methods

Thousands of years ago, a sun-drying method is used all over the world, even nowadays. In this method, the sun is directly exposed to the grape bunches which were spread over the ground or on a plastic sheet or different sizes of tray. Sun-drying process is the most cost-effective and requires less time to make raisins as compared to other traditional methods due to the penetration of solar radiation into the grape berries, which produces heat on the surface and in the core of the berries. As a result, this mechanism accelerates heat transfer and quickly evaporates the moisture content (Mahmutoglu, 1996). Conversely, insect infection, dust, and other environmental risks lead to a significant decline in quality (Bhat et al., 2006). Furthermore, the raisins that were subjected to direct as well as intense sun radiation (due to temperature variations) showed differences in color, appearance, and aroma (Pangavhane & Sawhney, 2002). Air-drying or shade drying is also known as the traditional method of grape drying. This method is most commonly used in China, India, and Australia, in which natural air is used as a principal source for drying (Pangavhane & Sawhney, 2002).

In the early research (Buttery et al., 1981, Ramshaw and Hardy, 1969), the raisin was prepared from TS grapes by sun-drying method and identified 38 and 40 VOCs, respectively. They found that the key VOCs were octanoic acid, nonanoic acid, (*E*)-2-octenal, (*E*)-2-decenal, nonanal, furfural, methyl furfural, 2-hydroxybutan-3-one and diacetyl. In a subsequent experiment, 32 VOCs were found in the must made from sun-dried Pedro Ximenez grapes. Among these VOCs, hexanoic acid, isobutanol, isoamyl alcohol, benzyl alcohol, 2-phenylethanol, *γ*-hexalactone, *γ*-butyrolactone, and 5-methylfurfural were significantly higher in sun-dried must as compared to fresh grapes must (Franco et al., 2004). Around 25 years later, Wang et al., 2017, Wang et al., 2017 detected 91 free- and bound-form volatile chemicals in sun-dried raisins from the three cultivars which include TS, CS, and ZS using the sophisticated technique “SPME”. They concluded that ZSRs performed exceptionally well because their floral OAV was much higher than the other two varieties, as well as, the fruity and green OAVs were also significantly differentiated. ZSRs' flowery and fruity aromas become prominent due to the presence of decanal, rose oxide, linalool, geraniol, and *β*-damascenone. In China, the dry and hot desert winds are used for raisins production in a specific ventilated room, which is made of clay bricks (Approx. 3 x 4 x 6–8 m^3^), along with several bricks-sized and cross-shaped openings in the walls (Fang et al., 2010). The air-dried raisins attained better color, appearance (Wang et al., 2016), and aroma quality (Wang et al., 2015) than sun-dried raisins, and also evade direct contact with sunlight.

#### 3.4.2 Modern technologies

With the rapid advancement in agricultural technologies and mechanization; modern drying techniques have been commonly used in developing countries for rapid, controlled, and high-quality production of raisins by using less labor.

Christensen (2000) reported that other modern technologies such as oven drying, microwave heating, freeze drying, and vacuum pulsed drying are used for raisins production. In recent years, microwave and oven drying have attained consideration owing to its less drying time, quick processing rate, instant, and accurate electronic control, as well as a clean heating process (Rattanadecho & Makul, 2016). In the microwave drying process, electromagnetic radiation is converted into thermal energy to vaporize the moisture from the grapes, whereas direct thermal energy is employed to dehydrate the food product in the oven drying process (Michailidis & Krokida, 2014). Furthermore, vacuum drying is a method in which fresh produce is dried under sub-atmospheric pressure and is magnificently used in raisins processing (Parikh, 2015). Recently, the infrared drying method has become more popular in the food industry due to its short drying time, producing the best final product, and the capacity to consume less energy. In addition, it is a more cost-effective method in comparison to microwave and vacuum drying techniques (Riadh et al., 2015). The main goal of these modern technologies is to obtain the best quality product by utilizing less energy and labor. The combination of these technologies can be helpful to achieve that goal.

The influence of several drying techniques (freeze-drying, oven-drying, microwave-dried, and shade-drying) on the volatile profile of strawberries was compared using the HS-SPME/GC–MS method, and 167 VOCs were characterized. The results revealed that shade air-dried, frozen, freeze-dried, and oven-dried (at 45 °C) strawberries retained more sweet and fruity, representing more than 68 % of the total aroma intensity. In contrast, the microwave-drying method showed a drastic loss of fruity esters (Abouelenein et al., 2021). Recently, a study was conducted on the changes in volatile organic chemicals, in which lemon flavedo was dried using integrated freeze drying (IFD), conventional freeze drying (CFD), and hot-air drying (AD). The findings indicated that IFD, followed by CFD and AD, preserved the most volatile aromas. Furthermore, CFD, IFD, and AD samples lost 56.07 mg/g, 47.92 mg/g, and 13.22 mg/g of total volatiles, respectively, compared with fresh samples (Hu et al., 2023). A series of research works have been done on the physical, physiological, and biochemical characteristics of raisins using modern drying techniques (Caglar et al., 2009, Kassem et al., 2010, Wang et al., 2017, Wang et al., 2017), but no one has looked at the impact of these techniques, particularly on aromatic components.

Accordingly, drying procedures have a significant impact on raisins' aromas. The high-temperature drying technique or sun-drying method did not retain the fresh aroma, but it produced a more roasted and fatty aroma. Contrarily, it was discovered that air-dried, shade-dried, and freeze-dried raisins preserved a fresh aroma and were also responsible for producing fruity, floral, and green aromas in raisins.

### 3.5 Storage

Raisins can be kept at room temperature for a few months without losing their taste, flavor, or color. The storage stability of raisins depends upon the room temperature, relative humidity (RH), and moisture level in raisins. The optimum range of RH for raisin storage is about 45–55 % (Bhat et al., 2006). However, to retain the quality for long-term storage, raisins can be kept in the refrigerator, as well as in a controlled atmosphere and modified atmosphere.

According to Kaack and Christensen (2010), storage conditions and packaging material significantly impacted the contents of VOCs present in tea. Javed et al. (2018) carried out a research study on the storage of air- and sun-dried raisins for 0, 3, 6, and 9 months in plastic and woven bags. They concluded that sun-dried raisins stored in plastic bags had greater total amounts of VOCs than those stored in woven bags. Furthermore, the fruity and floral aromas were noticeably affected by plastic bags during the storage periods (3, 6, and 9 months). Another study conducted on the storage of TSRs revealed that the fruity, floral, herbaceous, and roasted aromas improved during the storage period and became more compelling in 12 months (Javed et al., 2019).

## 4 Origin of raisin volatiles

**Fig. 3:**
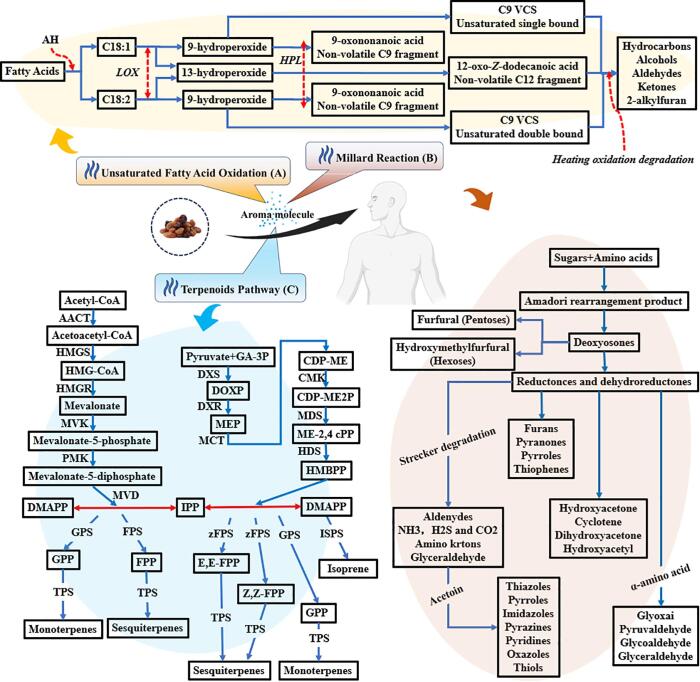
Proposed pathways for the generation of the raisin aroma.

Previous studies have reported that raisin VOCs are derived from different sources such as fresh grapes, unsaturated fatty acid auto-oxidation (UFAO), Maillard reactions, and carotenoids. A schematic diagram (Fig. 3) was proposed that illustrated the probable generation pathways of raisin VOCs based on previous research regarding metabolic aroma biosynthesis in grapes and wine.

### 4.1 Lipid oxidation

Lipid-derived VOCs are one of the major groups of food flavors. The consumer acceptability of foods was influenced as a result of the formation of VOCs by the process of lipid oxidation. Lipid oxidation is carried out in plant tissue by the enzyme at periods of maturity, growth, development, or tissue disturbance. In addition, it imparts a distinctive flavor to a variety of fruits and vegetables (Jelen & Wasowicz, 2011), including raisins (Javed et al., 2021). The fatty-derived compounds are also the source of fruity flavor, produced by enzymatic breakdown, α-oxidation, and *β*-oxidation in intact or disrupted fruits through the autoxidation of unsaturated fatty acid (Schwab & Schreier, 2002).

The oxidation process of unsaturated fatty acids is shown in Fig. 3A. The primary enzymes involved in this reaction included lipase, lipoxygenase (LOX), alcohol dehydrogenase (ADH), and hydroperoxide lyase (HPL). In terms of unsaturated fatty acids, TSRs and CSRs were found to have the highest concentrations of linoleic acid (C18:1) and linolenic acid (C18:2), which ranged from 1.6 to 2.2 ug/g (Wang et al., 2021). Both linoleic acid and linolenic acid are oxidized into 9- & 13-hydroperoxides by the action of LOX. The 13-hydroperoxides further oxidize to aldehyde compounds by HPL, as well as aldehyde compounds converted to alcohols by ADH (Stone et al., 1975).

Overall, 46 UFAO-VOCs were reported in different raisin cultivars (Table 3.), accounting for a significant portion of the raisin aroma (Bhouri et al., 2016, Buttery et al., 1981, Franco et al., 2004, Javed et al., 2018, Javed et al., 2019, Joulain and Fourniol, 1990, Ramshaw and Hardy, 1969, Wang et al., 2015, Wang et al., 2017, Wang et al., 2017). Wang et al. (2015) analyzed the raisin volatile chemicals in air-dried TSRs, CSRs, and FSRs and discovered that the main contributors among UFAO-derived compounds were hexanal, hexanoic acid, (*E,E*)-2,4-heptadienal, and 1-octen-3-ol. Furthermore, acidic compounds such as hexanoic acid (675 µg/L), heptanoic acid (198 µg/L), octanoic acid (182 µg/L), nonanoic acid (150 µg/L), decanoic acid (133 µg/L) and dodecanoic acid (137 µg/L) were leading compounds and significantly higher in ZSRs as compared to TSRs and CSRs (Wang et al., 2017, Wang et al., 2017). In another study, the volatile properties of three cultivars of raisins were examined while they were being stored for 0, 3, 6, and 9 months. Among 38 UFAO-derived volatile, the contents of heptanoic acid, octanoic acid, 1-hexanol, and 2,6-dimethyl-4-heptanone were plentiful (Javed et al., 2018). Additional 40 UFAO-VOCs were discovered in TS that had been air and sun-dried and stored for 0, 4, 8, and 12 months. In terms of UFAO-based volatile, heptanal, hexanal, pentanal, hexanoic acid, *n*-decanoic acid, and (*E,E*)-2,4-heptadienal had noticeably greater concentrations (Javed et al., 2019). As a result, numerous UFAO-derived compounds were detected in higher concentrations in various raisin cultivars, and these compounds are essential for raisin aroma. Later, Wang et al., 2020, Wang et al., 2020 used the advanced technique of “pulsed vacuum drying (PVD)” to make raisins from three different grape varieties, including TS, FS, and Munage, and the results showed that PVD-dried raisins were less brown and had a stronger fruity aroma and flavor when compared to shade drying. 17 UFAO-derived compounds out of 34 play a significant part in raisin aroma, and the outcomes prove that PVD is an intriguing substitute for producing top-notch raisins.

**Table 3:** Volatile compounds reported in raisins.

S/No	CAS No.	UFAO-derived Volatile compounds	Source	OTV (µg/L)	Aroma descriptor	Reported by
*UFAO-derived volatile compounds*						
1	**123**–**66-0**	Ethyl hexanoate	Ester	1**^3,7^**	Fruity, apple like **^a, g^**	J1, J2, W1, W2
2	**106**–**32-1**	Ethyl octanoate	Ester	194**^3^**	Apple, fruity, sweet **^b^**	B, J1, J2, R, T, W1, W2, W3
3	**111**–**11-5**	Methyl octanoate	Ester	200**^3^**	Fruity, citrus like **^a^**	J1, J2
4	**123**–**29-5**	Ethyl nonanoate	Ester	377**^3^**	Fruity, floral **^b^**	J1, J2
5	**96**–**48-0**	Butyrolactone	Ester	NF	NF	J, J1, J2, R, W1, W2
6	**123**–**25-1**	Diethyl succinate	Ester	500000**^3^**	Fruity, wine **^a, f^**	J, W1, W2
7	**112**–**30-0**	Methyl hexadecanoate	Ester	NF	NF	J2
8	**628**–**97-7**	Ethyl hexadecanoate	Ester	2000**^3^**	Wax **^a^**	J1, J2, W1, W2
9	**104**–**61-0**	*γ*-Nonalactone	Ester	30**^6^**	Coconut, peach **^a^**	J, J1, J2, W1, W2, W3
10	**110**–**62-3**	Pentanal	Aldehyde	12**^3,7^**	Fat, green **^g^**	J1, J2, R, W1, W2
11	**124**–**13-0**	Octanal	Aldehyde	0.7**^3,7^**	Honey, green, fatty **^g^**	B, B1, J1, J2, T, W1, W2
12	**124**–**19-6**	Nonanal	Aldehyde	1**^3,7^**	Green, fruity **^g^**	B1, J1, J2, W1, W2, W3
13	**66**–**25-1**	Hexanal	Aldehyde	4.5**^3,7^**	Green **^g^**	B1, J1, J2, R, W1, W2, W3
14	**111**–**71-7**	Heptanal	Aldehyde	3**^3,7^**	Dry fish, smoky **^g^**	B1, J1, J2, W1, W2
15	**6728**–**26-3**	(*E*)-2-Hexenal	Aldehyde	17**^3,7^**	Green **^g^**	B1, J1, J2, R, W1, W2, W3
16	**18829**–**55-5**	(*E*)-2-Heptenal	Aldehyde	13**^3,7^**	Fatty, soapy, tallow **^g^**	B1, J1, J2, W1, W2, W3
17	**2548**–**87-0**	(*E*)-2-Octenal	Aldehyde	3**^3^**	Green, fatty, nut **^g^**	B1, J1, J2, W1, W2
18	**18829**–**56-6**	(*E*)-2-Nonenal	Aldehyde	0.08**^3^**	Green, fat **^a^**	B1, J, W1, W2
19	**112**–**31-2**	Decanal	Aldehyde	0.1**^3^**	Sweet, citrus, green **^e^**	B, B1, F, J1, J2, W2
20	**4313**–**03-5**	(*E,E*)-2,4-Heptadienal	Aldehyde	49^4^	Fatty, hay **^b^**	B, B1, J1, J2, W1, W2
21	**5910**–**87-2**	(*E,E*)-2,4-Nonadienal	Aldehyde	0.09**^3^**	Fatty, oily **^b^**	B1, J1, J2, W1, W2
22	**25152**–**84-5**	(*E,E*)-2,4-Decadienal	Aldehyde		Seaweed **^a^**	B1
23	**74**–**41-20**	1-Pentanol	Alcohol	4000	Balsamic, almond ^c^	J2, W1, W2, W3
24	**111**–**27-3**	1-Hexanol	Alcohol	2500**^3^**	green **^b^**	J1, J2, W1, W2, W3
25	**928**–**96-1**	(*Z*)-3-Hexen-1-ol	Alcohol	100**^3,5^**	Fruity, green **^b^**	F, J1, J2
26	**3391**–**86-4**	1-Octen-3-ol	Alcohol	1**^3,7^**	Mushroom, fruity **^b, g^**	B, B1, F, J1, J2, W1, W2
27	**111**–**70-6**	1-Heptanol	Alcohol	1000**^3^**^,^**^5^**	Grape, sweet **^c^**	J1, J2, W1, W3
28	**104**–**76-7**	2-Ethyl-1-hexanol	Alcohol	270000**^3^**	Floral, sweet fruity **^c^**	J1, J2, W1, W2
29	**111**–**87-5**	1-Octanol	Alcohol	130**^2,3^**	Citrus, rose **^c^**	B1, J1, J2, W1, W2, W3
30	**123**–**96-6**	2-Octanol	Alcohol	NF	Mushroom, fat **^a^**	B1, J1, J2, W2
31	**18409**–**17-1**	(*E*)-2-Octen-1-ol	Alcohol	18^5^	Fatty, rancid **^b^**	J1, J2, W1, W2, W3
32	**143**–**08-8**	1-Nonanol	Alcohol	50**^3^**	Floral **^b^**	B1, J1, J2, W1, W2
33	**628**–**99-9**	2-Nonanol	Alcohol	58^4^	Fruity, green **^d, f^**	J1, J2
34	**109**–**52-4**	Pentanoic acid	Acid	3000**^3^**	Sweet	J1, J2, W1, W2
35	**142**–**62-1**	Hexanoic acid	Acid	3000**^3,7^**	Raincid, chees, fatty **^g^**	B, B1, F, J, W1, W2, W3
36	**111**–**14-8**	Heptanoic acid	Acid	3000**^3^**	Sweety, cheesy **^f^**	B1, J1, J2, W1, W2, W3
37	**124**–**07-2**	Octanoic acid	Acid	3000**^3,7^**	Raincid, chees, fatty **^g^**	B1, F, J1, J2, W1, W2, W3
38	**110**–**05-0**	Nonanoic acid	Acid	3000**^3,7^**	Raincid, chees, fatty **^g^**	B, B1, J1, J2, W1, W2, W3
39	**334**–**48-5**	Decanoic acid	Acid	10000**^3^**	Fatty, raincid **^a^**	J1, J2, W1, W2, W3
40	**334**–**48-5**	*n*-Decanoic acid	Acid	10000**^3^**	Fatty, raincid **^a^**	J2, W3
41	**143**–**07-7**	Dodecanoic acid	Acid	10000^3^	Dry, metallic f	J2, W, W1
42	**79**–**31-2**	2-Methyl-propanoic acid	Acid	8100^3^	Rancid, butter, cheese ^a,c^	J1, J2, W
43	**123**–**51-3**	3-Methyl-1-butanol	Acid	3003	Malt, whiskey ^a^	J, J1, J2, J3, W1, W2
44	**108**–**83-8**	2,6-Dimethyl-4-heptanone	Ketone	NF	NF	J1, J2, W1, W2
45	**1669**–**44-9**	3-Octen-2-one	Ketone	NF	Green, fruity **^b^**	B, J1, J2
46	**3777**–**69-3**	2-Pentyl furan	Furan	6**^3,7^**	Fruity, green, sweet **^g^**	B, B1, J1, J2, W1, W2

***MR-derived volatile compounds***						
01	**100**–**52-7**	Benzaldehyde	Aldehyde	3503^7^	Sweet, fruity ^g^	F, J2, J3, R, W1, W2
02	**122**–**78-1**	Phenylacetaldehyde	Aldehyde	43^7^	Flowery, rose ^g^	J1, J2, J3
03	**100**–**51-6**	Benzyl alcohol	Alcohol	10000^3,7^	Fruity, sweet ^g^	F, J2, R, W1, W2
04	**431**–**03-8**	2,3-Butanedione	Ketone	100^8^	Butter ^a^	J2, J3, W1, W2
05	**13925**–**03-6**	2-Ethyl-6-methyl, pyrazine	Pyrazine	NF	Nutty, grassy ^b^	J2, J3, W1, W2, W3
06	**108**–**50-9**	2,6-Diethyl, pyrazine	Pyrazine	6^6^	Roasted, nutty ^b^	J2, J3, W1, W2
07	***13360***–***65-1***	3-Ethyl-2,5-dimethylpyrazin	Pyrazine	1^3^	Nutty roasted, woody ^e^	J, J2, J3, W1, W2
08	**98**–**01-1**	Furfural	Furan	3000^3^	Sweet, almond ^b,c^	B1, F, J2, J3, R, W1, W2, W3
09	**1192**–**62-7**	2-Acetylfuran	Furan	10,000^3^	Smoky, sweet ^b^	J2, J3, W1
10	**620**–**02-0**	5-Methyl-2-furfural	Furan	NF	Warm, burnt, spicy ^b^	F, B1, J, J1, J2, J3, W1, W2
11	**67**–**47-0**	5-Hydroxymethyl-2-furaldehyde	Furan	NF	NF	J2, J3

***Terpenoids-derived volatile compounds***						
01	**77**–**53-2**	Cedrol	Terpene		Cool, camphor ^f^	K
02	**78**–**70-6**	Linalool	Terpene	6^3,6^	Fruity, sweet, grape ^b,e^	B, K, W1, W2, W3, J1, J2, J3
03	**80**–**56-8**	*α*-Pinene	Terpene		Pine, resinous ^f^	K
04	**98**–**55-5**	*α*-terpineol	Terpene	350^3^	Floral, sweet ^g^	B1, F, J2, J3, K, R, W1, W3
05	**99**–**83-2**	Phellandrene	Terpene		Terpene, fruity, minty ^f^	K
06	**99**–**85-4**	*γ*-Terpinene	Terpene	1000^3,7^	Fruity, lemon like ^g^	J1, K, W2
07	**106**–**22-9**	*β*-Citronellol	Terpene	62^5^	Sweet, citrus like ^f^	J1, J2, J3, W1, W2
08	**106**–**24-1**	Geraniol	Terpene	40^3,7^	Floral, rose, citrus ^g^	J1, J2, J3, K, W1, W2, W3
09	**106**–**25-2**	Nerol	Terpene	300^3,7^	Flower, grass, floral ^g^	J1, J2, J3, K, W1, W2
10	**123**–**35-3**	*β*-Myrcene	Terpene		Green burning, green	K
11	**127**–**91-3**	*β*-Pinene	Terpene		Woody, resinous ^f^	K
12	**138**–**86-3**	Limonene	Terpene	10^3,7^	Fruity, lemon ^a,g^	J1, J3, W2
13	**459**–**80-3**	Geranic acid	Terpene	40^7^	Green ^g^	J1, J2, J3, K, W1, W2, W3
14	**460**–**01-5**	Cosmene	Terpene	NF	NF	J1, J3
15	**502**–**99-8**	*α*-Ocimene	Terpene	NF	Fruity ^f^	J1, J3,
16	**527**–**84-4**	*o*-Cymene	Terpene	NF	Citrus, green ^f^	J1, J2, J3, K
17	**562**–**74-3**	4-Terpineol	Terpene		Flowers, nutmeg, moldy ^f^	K
18	**586**–**62-9**	Terpinolene	Terpene	200^3,7^	Piney ^g^	B1, J1, J2, J3, K, R, W2, W3
19	**586**–**81-2**	*γ*-terpineol	Terpene		NF	W2
20	**673**–**84-7**	Alloocimene	Terpene		NF	W2
21	**689**–**67-8**	Geranylacetone	Terpene	60^3^	Rose, floral ^b,f^	B1, J, J1, J2, J3, W1, W2
22	**1679**–**51-2**	3-Cyclohexene-1-methanol	Terpene	330^7^	Floral, sweet ^g^	J1
23	**1786**–**08-9**	Nerol oxide	Terpene	3000^7^	Oil, flowery ^g^	J1, J2, J3, K, W1, W2
24	**4610**–**11-1**	(*E*)-Rose oxide	Terpene	NF	Rose ^f^	K
25	**5392**–**40-5**	(*E*)-Neral	Terpene	1000^7^	Fruity ^g^	J1, J2, K, W1
26	**5989**–**27-5**	*d*-Limonene	Terpene	NF	Fruity, lemon ^f^	K
27	**7212**–**44-4**	*E*-Nerolidol	Terpene	NF	Rose, apple, gree, waxy, woody ^f^	K
28	**14009**–**71-3**	*cis*-Pyran linalool oxide	Terpene	NF	Floral, fruity, citrus ^a^	B, J1, J3, W2
29	**16409**–**43-1**	Rose oxide	Terpene	0.5^3,7^	Rose, floral ^a,g^	J1, J3, W2
30	**19894**–**97-4**	Myrtenol	Terpene	NF	Flowery, mint ^f^	K
31	**20053**–**88-7**	Hotrienol	Terpene	110^7^	Fresh, floral, fruity ^g^	J1, J2, J3, K, W1, W2, W3
32	**23726**–**91-2**	*β*-Damascenone	Terpene	0.09^6^	Sweet, floral, fruity ^g^	J1, J2, J3, K, W1, W2, W3
33	**29548**–**14-9**	*p*-Menth-1-en-9-al	Terpene	NF	NF	J1, J2, J3, W2
34	**33081**–**36-6**	Lilac alcohol	Terpene	NF	NF	JI, J3, W1, W2
35	**40716**–**66-3**	Nerolidol 2	Terpene	NF	NF	J2, J3
36	**52462**–**29-0**	*p*-Cymene	Terpene	11.4a	Citrus, citric, carrot solvent	W1, W2
37	**57396**–**75-5**	3,4-Dimethyl-2,4,6-octatriene	Terpene	NF	NF	J1, J2
38	**61142**–**36-7**	3-Ethyl-2-methyl-1,3-hexadiene	Terpene	NF	NF	W1

***Carotenoids-derived volatile compounds***						
01	**14901**–**07-6**	*β*-Ionone	Ester	0.007^3,7^	Balsamic, rose ^g^	J1, J2, J3
02	**1569**–**60-4**	6-Methyl-5-hepten-2-ol	Alcohol	2000^3^	Fruity, sweet ^g^	J1, J2, J3, W1, W2
03	**110**–**93-0**	6-Methyl-5-hepten-2-one	Ketone	50^3^	Sweet, fruity ^f^	J1, J2, J3, W1, W2, W3
04	**1604**–**28-0**	6-Methyl-3,5-heptadiene-2-one	Ketone	380^3^	Warm, spicy ^b^	B1, J1, J2, J3, W1

***Other volatile compounds***						
01	**101**–**97-3**	Phenethyl acetate	Ester	650^3^	Fruit, sweet ^a^	J1, K, W1, W2
02	**110**–**38-3**	Ethyl decanoate	Ester	200^4^	Fruity, apple ^b^	B, J1, W2
03	**118**–**61-6**	Ethyl salicylate	Ester	NF	Green, mint ^a^	J1, J2, J3
04	**119**–**36-8**	Methyl salicylate	Ester	40^3,7^	Green, pine ^a,g^	J, J1, J3, W1, W2
05	**140**–**11-4**	Benzyl acetate	Ester	Nf	Floral, fruity, sweet ^a^	J2, W1, W2
06	**141**–**78-6**	Ethyl Acetate	Ester	5000^3,7^	Fruity, solvent ^g^	J1, J2, J3, W1, W2, W3
07	**64**–**17-5**	Ethyl alcohol	Alcohol	100000^3^	Sweet ^a^	B, J1, J2, J3, R, W2
08	**71**–**36-3**	1-Butanol	Alcohol	500^3^	Fruity, floral ^b^	J1, J2, J3
09	**556**–**82-1**	3-Methyl-2-buten-1-ol	Alcohol	NF	NF	J, J1, J2, J3, W1, W2
10	**60**–**12-8**	Phenylethyl alcohol	Alcohol	1100^3^	Floral, rose, honey ^g^	B, J1, J2, J3, W1, W2, W3
11	**64**–**19-7**	Acetic acid	Acid	60000^5^	Vinegar d	J1, J3
12	**112**–**05-0**	Nonanoic acid	Acid	3000^3^	Green, fat ^a,f^	B, B1, J, J1, J2, J3, W1, W2, W3
13	**149**–**57-5**	2-Ethylhexanoic acid	Acid	NF	NF	J1, J2, J3, W1
14	**98**–**86-2**	Acetophenone	Ketone	65^3^	Sweet, flowery ^a^	B, J, J1, J2, J3, R, W1, W2
15	**513**–**86-0**	Acetoin	Ketone	800^3^	Buttery, cream ^a^	J, J1, J2, J3, R, W1, W2, W3
16	**91**–**20-3**	Naphthalene	Benzene	NF	Tar ^a^	J1, J2, J3, W1, W2, W3
17	**91**–**57-6**	2-Methyl-naphthalene	Benzene	NF	NF	J1, J2, J3, W1, W2, W3
18	**100**–**42-5**	Styrene	Benzene	730^3^	Balsamic, gasoline ^a^	J1, J2, J3
19	**108**–**88-3**	Toluene	Benzene	NF	Paint ^a^	J1, J2, J3, W2
20	**108**–**95-2**	Phenol	Phenol	5900^3^	Medicinal ^a,d^	J1, J2, J3, W1, W2
21	**7786**–**61-0**	4-Vinylguaiacol	Phenol	3^3^	spicy, curry ^a,f^	J1, J2, J3, W3

**Volatile compound in raisins reported by:**

^B1^ (Buttery et al., 1981).

^B^ (Bhouri et al., 2015).

^F^ (Franco, Peinado, Medina, & Moreno, 2004).

^J^ (Joulain & Fourniol, 1990).

^K^ (Karakus et al., 2023).

^R^Ramshaw & Hardy, 1969).

^W1^ (Wang et al., 2015).

^W2^ (Wang et al., 2017).

^W3^ (Wang et al., 2020).

^J1^ (Javed et al., 2018).

^J2^ (Javed et al., 2019).

^J3^ (Javed et al., 2021).

**OTV** (Odor Threshold values; Parts per Billion, µg/L) **in water were reported by:** (**^1^**Ho, Zheng, & Li, 2015; **^2^**Jiang & Zhang, 2010; **^3^**Leffingwell & Associates, 2004; **^4^**Nan et al., 2013; **^5^**Qian & Wang, 2005; **^6^**Wang et al., 2017; **^7^**Wu et al., 2016; **^8^**Welke et al., 2014).

**Aroma descriptors were obtained from** “Flavornet and human odor space” (**^a^**https://www.flavornet.org/flavornet.html), the LRI and odor data base (**^b^**https://www.odour.org.uk/odour/index.html) and from reported literature (**^c^** Jiang & Zhang, 2010; **^d^** Qian & Wang, 2005; **^e^**Wang et al., 2017, Wang et al., 2017; **^f^** Welke et al., 2014; **^g^** Wu et al., 2016, **^f^**Karakus et al., 2023).

“NF” indicated not found.

### 4.2 Maillard reaction

The Maillard reaction (MR), particularly in heated/dried foods, has a significant effect on food quality. The nutritional value of food is impacted by browning due to MR, which may also result in the production of antioxidant molecules and a distinct flavor of food (Van Boekel, 2006).

MR is usually classified into three (3) stages (Fig. 3B). The first stage starts with a condensation process among reducing sugar and amino acids, leading to the formation of Amadori (if aldose is reducing sugar) or Heyns product (ketose reducing sugar). The second stage starts from the Heyns or Amadori product that converts into sugar fragmentation and discharge of the amino group. In the last stage, all kinds of dehydration, cyclization, fragmentation, and polymerization reactions are carried out, in which amino acid again takes part. In the case of flavor formation, Strecker degradation is of utmost importance. The several possible reaction pathways depended on temperature, pH, and kind of sugar, amino acid, and protein.

A total of 11 VOCs, including 2,3-butanedione, 5-hydroxymethyl-2-furaldehyde, 2-ethyl-6-methyl, pyrazine, 5-methyl-2-furfural 2,6-diethyl, pyrazine, 3-ethyl-2,5-dimethylpyrazin, furfural, 2-acetylfuran, benzaldehyde, benzyl alcohol, and phenylacetaldehyde were produced by MR (Table 3.) in different raisin cultivars (Javed et al., 2018, Javed et al., 2019, Wang et al., 2015, Wang et al., 2017, Wang et al., 2017). Sun-dried raisins were shown to contain higher concentrations of MR-derived compounds, such as pyrazines (2,6-diethyl pyrazine and 3-ethyl-2,5-dimethyl pyrazine), furans (2-pentylfuran), and benzene-acetaldehyde which significantly contributed to the roasted, fruity and floral aromas (Wang et al., 2015, Wang et al., 2017, Wang et al., 2017). Among 10 identified MR compounds, benzyl alcohol, 2,3-butanedione, and 2-acetylfuran were the leading compounds in the pre-treated sun- and air-dried raisin during the period of storage. They concluded that CSRs were more MR-compounds rich variety as compared to TSRs and FSRs. Furthermore, the concentration of 2,6-diethyl pyrazine, 5-ethyl-2,3-dimethylpyrazin, 2-acetylfuran, and 5-methyl-2-furfural in pre-treated sun-dried raisin was increased during storage (Javed et al., 2018). Another study was carried out to identify the effect of the drying method (sun- and air-dried), packaging material (plastic and woven bag), and storage period (0, 4, 8, and 12) on the free- and bound-form VOCs in Thompson seedless raisin. In this study, 2,3-butanedione, furfural, and phenylacetaldehyde were more prevalent among MR-derived compounds. Overall, the content of MR compounds was significantly higher in the sun-dried method and was increased with the storage period (Javed et al., 2019). According to these findings, the MR compounds were specific to the grape variety and concentrated under the sun drying procedure, as well as their concentration was raised with the storage period.

### 4.3 Terpenoids-derived volatile compounds

Terpenoids are the most prevalent and structurally diverse secondary metabolites in plants. They are vital to plant survival because they facilitate both direct and indirect defense mechanisms, attract pollinators, and boost various interactions between the plants and the environment. They are considered one of the most important classes that contribute to fruits' characteristically fruity and floral aromas (Macleod & de Troconis, 1982) and play a significant role to produces aromas in raisins (Javed et al., 2019, Wang et al., 2017, Wang et al., 2017). Likewise, they can be synthesized via two different pathways: the 2-C-methyl-*d*-erythritol-4-phosphate (MEP) and the mevalonate (MVA) pathway. MEP is sometimes referred to as the 1-deoxy-*d*-xylulose-5-phosphate (DXP) pathway. Fig. 3C briefly describes both distinct synthesis pathways and their corresponding processes.

Until now, there have been 38 terpenoid-derived VOCs characterized in various raisin varieties that were dried via different techniques (Table 3.). Thirteen of the 15 terpenoids were discovered for the first time in three seedless grape varieties—TS, FS, and CS—that were air-dried. In the free-form, geraniol, geranic acid, and nerol all achieved comparatively high concentrations (149–237 µg/L), however in the bound form, nerol, geraniol, *β*-damascenone, geranic acid, and neral were more abundant. They came to the conclusion that the red varieties (CS > FS) have greater terpenoids than the green one (TS) (Wang et al., 2015). Later, in follow-up research, Wang et al., 2017, Wang et al., 2017 found 24, 17, and 13 terpenoids in sun-dried cultivars ZS, CS, and TS, respectively. The highly concentrated terpenoid components in the sun-dried raisin, notably the ZS species, were nerol oxide (156 ± 1 µg/L), linalool (184 ± 10 µg/L), nerol (206 ± 1 µg/L), geraniol (558 ± 58 µg/L), hotrienol (1071 ± 15 µg/L), and geranic acid (1091 ± 31 µg/L). The same research group conducted a study on pre-treated air- and sun-dried raisins that were stored for 0, 3, 6, 9, and 12 months at room temperature. Amongst 21 terpenoid compounds, geraniol, γ-terpineol, and linalool were the three main terpenoids that give muscat flavor, and their concentrations declined with storage. Further, the pre-treated air-dried raisins were significantly more concentrated with nerol, geraniol, 3-cyclohexane-1-methanol, terpinolene, and linalool than pre-treated sun-dried raisins (Javed et al., 2018). Another study reported 15 terpenoids from sun- and air-dried raisins that were stored in different types of packaging. They reported that the air-dried raisins contained higher hotrienol, nerol oxide, and geraniol content, as well as, these content were increased with storage duration (Javed et al., 2019). In the most recent study on the Turkish raisin known as “Gök Üzüm,” 24 terpenoid components were detected in both the potassium carbonate and wood ash solutions. The contents of α-pinene, *d*-limonene, phellandrene, *β*-myrcene, *o*-cymene, *γ*-terpinene, terpinolene, (*E*)-rose oxide, (*Z*)-rose oxide, linalool, 4-terpineol, geranial, *E*-nerolidol, nerol, citronellol, and cedrol were greater in Gök Üzüm raisins made from grapes dipped into the wood ash solution than those grapes dipped into potassium carbonate solution (Karakus et al., 2023).

### 4.4 Carotenoids-derived volatile compounds

Carotenoids are plentiful in volatile apocarotenoids' precursors that provide aromas to fruits, flowers, and vegetables (Saini et al., 2015, Zheng et al., 2021). Moreover, carotenoids derived compounds play a significant role in the flavor and fragrance of both grapes and raisins (Javed et al., 2018, Wang et al., 2017, Wang et al., 2017). There were only four compounds were identified in different raisin varieties (Table 3.). Using the HS-SPME approach, Wang et al. (2015) reported an abundance of 6-Methyl-5-hepten-2-one and 2,6-dimethyl-4-heptanone for the first time in three air-dried raisins varieties. According to a study on raisin pretreatments, the pre-treated air-dried approach generated a higher content of sulcatol in TS raisins; 6-methyl-5-hepten-2-ol, 6-methyl-5-hepten-2-one, and geranylacetone in CS raisins. Furthermore, as the storage duration prolonged correspondingly increased the concentrations of geranylacetone and 6-methyl-3,5-heptadiene-2-one (Javed et al., 2018). In a different study, it was determined that air-dried raisins had more 6-methyl-5-hepten-2-one content than sun-dried raisins (Javed et al., 2018).

## 5 Odor Activity Value

The Odor Activity Value (OAV) has been commonly utilized in order to identify the active VOCs that contribute significantly to the aroma profile (Zhu et al., 2018). OAV, also known as an odor value or odor unit, is an assessment of odor intensity based on the ratio of a volatile compound's concentration to its odor threshold value. The reported aroma compounds with OAVs > 1 from different studies were generally categorized into six groups based on their sensory characteristics: floral, fruity, green, roasted, fatty, and chemical aromas.

**Fig. 4:**
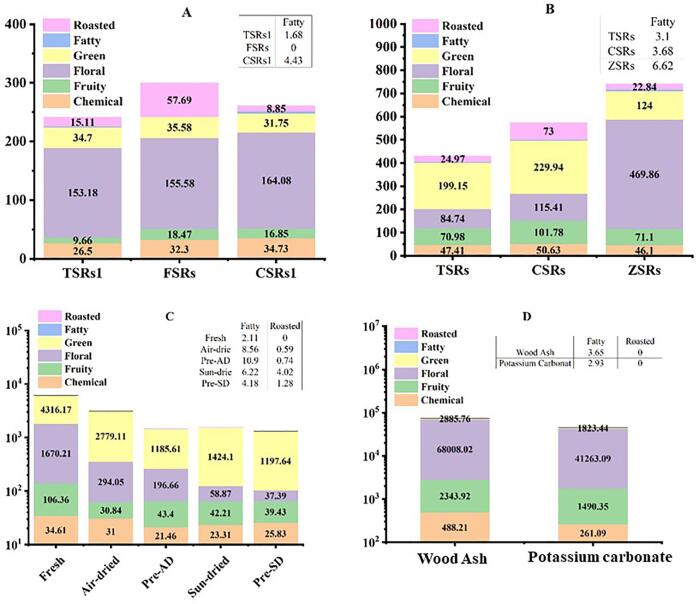
OAVs in different studies. A (Three raisin varieties dried by air); B (Three raisin varieties dried by air); C (Comparison of fresh, dried, and pre-treated raisin); D (Wood ash and potassium carbonate as a pre-dipping solution).

The 20 aromas active compounds were recognized as OAVs in air-dried TSRs, CSRs, and FSRs which made a significant contribution to raisin aromas. Among these, *β*-damascenone was the most prominent compound that had a higher OAV in all three varieties. A characteristics comparison of the three varieties revealed that the fruity, floral, green, and chemical OAVs were almost the same, whereas the roasted OAVs had higher in FSRs, followed by TSRs and CSRs with 57.69, 15.11, and 8.85 (Fig. 4A), respectively (Wang et al., 2015). A follow-up study used a traditional sun-drying procedure to yield 25 odor-active compounds from three grape varieties with varying levels of flavor intensity. Overall, CSRs were the richest flavoring varieties because it was having the potent fragrance of fruity, green, and roasted aromas as compared to TSRs and ZSRs (Fig. 4B). Rose oxide is a VOC that contains OAVs ≥ 1 and is responsible for the significant floral aroma in ZSRs. Besides that, hexanal and 3-ethyl-2,5-dimethylpyrazin mainly accounted for the roasted and green aromas, respectively (Wang et al., 2017). These two experiments were carried out by our research team utilizing the same procedure for volatile organic compound (VOC) extraction and identification. Therefore, comparison analysis revealed that sun-drying is more effective than air-drying based on CSRs and TSRs.

In order to provide additional evidence, Javed et al. (2021) carried out a comparison study on aroma compounds that are changed from fresh grapes to raisins by drying, both with and without pre-treatment solutions (lipid, KOH, and Na_2_CO_3_). Out of the 30 odor-active compounds, they also found that the predominant contributors to floral and fruity aromas were *β*-damascenone, while the primary contributors to green flavors were hexanal and (*E,E*)-2,4-nonadienal. Further, results concluded that the air-dried procedure retained the green, floral, and chemical fragrances, whereas the sun-dried method produced more roasted and fatty odors (Fig. 4C). The roasted aroma of grapes is brought about by the high temperature under the sun-drying process, which alters their green scent. In another research study, the pre-drying process for Gök Üzüm raisins was carried out using wood ash and potassium carbonate. The main odor-active compounds were *β*-damascenone (fruity), *β*-ionone (floral), rose oxide (floral), and cedrol (chemical), and the OAVs of these compounds were higher in the raisin treated with wood ash than in the raisin treated with potassium carbonate (Supplementary Table 1). Therefore, it was speculated that the wood ash pre-treatment provided Turkish raisins with stronger fruity, floral, green, and chemical fragrance (Fig. 4D) (Karakus et al., 2023).

**Fig. 5:**
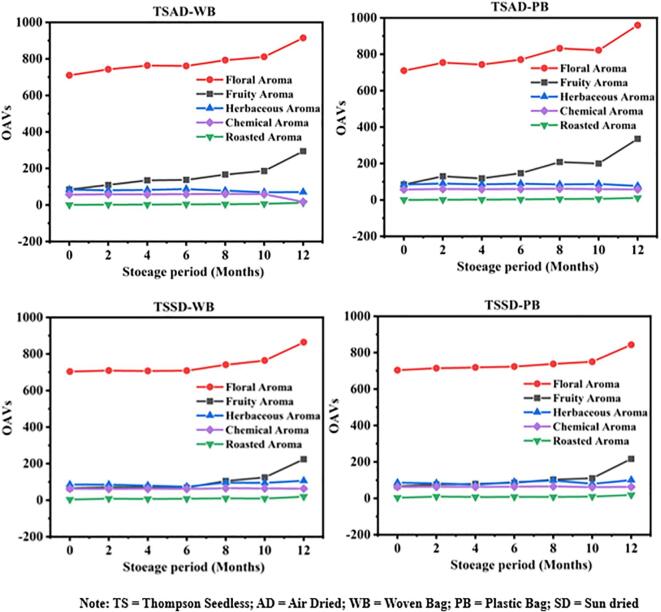
Effect of air-and sun-dried method on the active aromas of raisins. A1 (TSAD-WB); A2 (TSAD-PB); B1 (TSSD-WB); B2 (TSSD-PB).

Furthermore, the VOC changes experienced by air- and sun-dried TSRs during the storage were monitored. Similar patterns of aroma changes were seen with both treatments when the raisins were stored in plastic and woven bags (Fig. 5). After 0 to 10 months of storage, the OAVs for fruity and flowery fragrances were found to be the same in sun-and air-drying; however, after 12 months, they were shown to be more substantial (Fig. 5). As per the findings, air-drying preserved the fragrant qualities of fruits and flowers, but sun-drying produced a more roasted aroma in plastic bags as compared to woven bags (Javed et al., 2019).

## 6 Conclusion

Raisins are an excellent fruit selection for an individual's diet due to their reasonable price, accessibility, nutrition, and convenience. Raisin processing should be emphasized because the sensory quality of the final product has a significant impact on customer satisfaction. Up to now, over 120 volatile organic compounds (VOCs) have been documented; these are primarily extracted using a highly sophisticated method called solid phase microextraction (SPME). Further raisin aromas were influenced by various factors such as grape varieties, harvesting period, climate, drying process, storage, and extraction methodology. Different extraction methods were utilized to find the VOCs in raisins; the most successful approach was SPME. Moreover, it was greatly influenced by the drying process; high-temperature drying method or sun-drying process did not preserve the fresh aroma, instead, it resulted in a more roasted and fatty flavor. In contrast, it was found that air-drying, shade-drying, and freeze-drying methods maintain floral and green aromas. Regarding raisin cultivars, the red cultivars (CS and FS) were richest in green, fruity, and roasted fragrances, and had higher levels of terpenoids than the green variety (TS). Yet, there is no study regarding the effect of modern drying technology on raisin aromas. Therefore, future studies are needed in order to optimize modern technology to meet stringent raisin quality in terms of aromas. The consequences of modern drying techniques on the aromas of raisins have not yet been studied. Therefore, further research is necessary to optimize modern drying technology to meet the high standards for raisin quality in terms of fragrances.

## CRediT authorship contribution statement

**Hafiz Umer Javed:** Writing – original draft, Conceptualization. **Yuan-sen Liu:** Software, Formal analysis, Data curation. **Jun-guang Hao:** Supervision. **Faisal Hayat:** Writing – review & editing, Investigation. **Murtaza Hasan:** Writing – review & editing, Investigation, Formal analysis.

## Declaration of competing interest

The authors declare the following financial interests/personal relationships which may be considered as potential competing interests: Hafiz Umer Javed reports was provided by Beibu Gulf University. Hafiz Umer Javed reports a relationship with Beibu Gulf University that includes: employment and funding grants. Hafiz Umer Javed has patent NA pending to NA. we dont have any conflict If there are other authors, they declare that they have no known competing financial interests or personal relationships that could have appeared to influence the work reported in this paper.

## Data Availability

Data will be made available on request.
