# Supplementary figures and images for: Aromatic Perspectives: An In-Depth review on Extracting, influencing Factors, and the origins of raisin aromas
Source: Food Chem X. 2024 Mar 15;22:101285. doi: 10.1016/j.fochx.2024.101285 (PMC10973804; doi:10.1016/j.fochx.2024.101285)

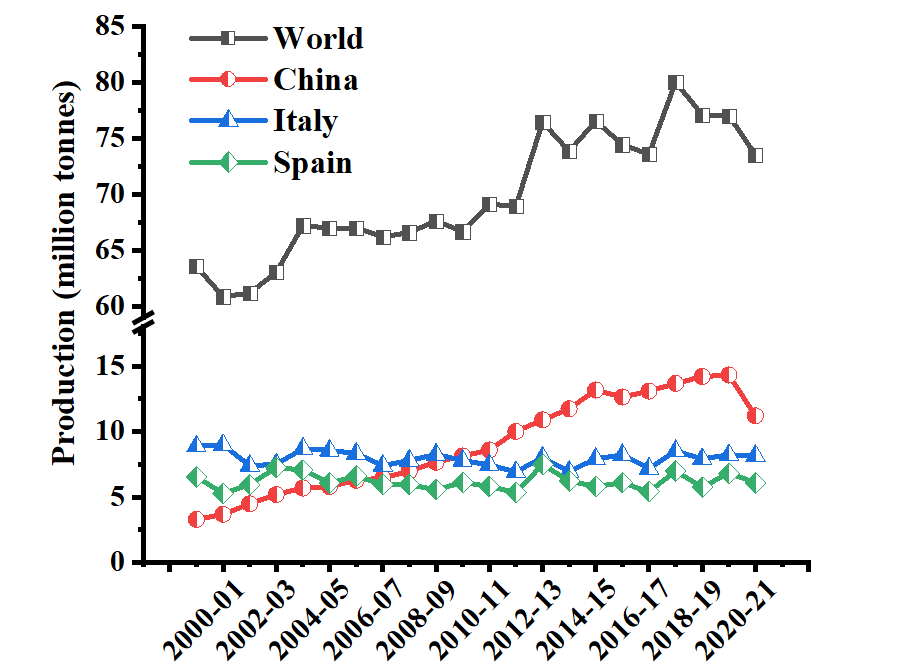

Supplement: Supplementary data 1 [file mmc1.zip › Supplementary Fig. 1.png]
